# Sociodemographic Variation of Caries Risk Factors in Toddlers and Caregivers

**DOI:** 10.1155/2010/593487

**Published:** 2010-09-23

**Authors:** G. J. Eckert, R. Jackson, M. Fontana

**Affiliations:** ^1^Division of Biostatistics, Indiana University School of Medicine, Indianapolis, IN 46202, USA; ^2^Department of Preventive and Community Dentistry, Indiana University School of Dentistry, Indianapolis, IN 46202, USA; ^3^Department of Cariology, Restorative Sciences and Endodontics, University of Michigan School of Dentistry, Ann Arbor, MI 48109-1078, USA

## Abstract

*Objectives*. Dental caries is the most common chronic childhood disease, with numerous identified risk factors. Risk factor differences could indicate the need to target caregiver/patient education/preventive care intervention strategies based on population and/or individual characteristics. The purpose of this study was to evaluate caries risk factors differences by race/ethnicity, income, and education. *Methods*. We enrolled 396 caregiver-toddler pairs and administered a 105-item questionnaire addressing demographics, access to care, oral bacteria transmission, caregiver's/toddler's dental and medical health practices, caregiver's dental beliefs, and caregiver's/toddler's snacking/drinking habits. Logistic regressions and ANOVAs were used to evaluate the associations of questionnaire responses with caregiver's race/ethnicity, income, and education. *Results*. Caregivers self-identified as Non-Hispanic African-American (44%), Non-Hispanic White (36%), Hispanic (19%), and “other” (1%). Differences related to race/ethnicity, income, and education were found in all risk factor categories. *Conclusions*. Planning of caregiver/patient education/preventive care intervention strategies should be undertaken with these caries risk factor differences kept in mind.

## 1. Introduction

According to the first-ever U.S. Surgeon General's report on oral health published in May 2000 [1], dental caries is the most common chronic childhood disease. In addition, most (75%–80%) of the caries in children in this country occur in a small segment of the population (20%–25%) [2], and this problem is particularly prevalent in minorities and immigrants and lower-income children [3]. Unfortunately, the funds to provide preventive care to all children who are either from a minority population or the lower SES levels are simply not available. Therefore, it is reasonable to attempt to identify those at highest risk in these populations and concentrate what limited financial and manpower resources are available in these “highest of the high” [4]. Additionally, the cultural, and behavioral determinants of disease and the barriers to access dental health services in these populations may be dissimilar to those in other social groups [5]. Understanding these differences may eventually influence different preventive strategies and alternative ways to help health providers to communicate with these groups in order to enhance health related behaviors and conditions. Therefore, identifying a child's level of risk for development of dental caries and the reasons behind it are necessary first steps in managing dental caries. 

Risk-based prevention and disease management have been recognized as the cornerstones of modern caries management [6–8], especially in young children [8–10]. The fact that the existence of past restorations is one of the greatest indicators of risk for the development of new caries lesions [9, 11] only proves that the act of surgically treating the caries lesion does little to reduce the risk of developing the next lesion, generally makes no significant difference to bacterial loading, nor on the enactment of self-promoting health behaviors such as brushing one's teeth [12–14]. The etiology of the dental caries process is multifactorial in nature and involves a combination of factors including diet, a susceptible host, and microflora, which interplay with a variety of social, cultural and behavioral factors. Additionally, most young children appear to acquire some cariogenic microorganisms (i.e., mutans streptococci-MS) from their mothers or primary caregivers [15]. Transmission happens through saliva and can be affected among other variables by the frequency of the contact (e.g., sharing of food and utensils, kissing, etc.), which could have cultural and behavioral determinants and, therefore, may vary among different ethnic and cultural population groups. Because of the multifactorial nature of the dental caries disease process, and the fact that the disease is very dynamic, but not continuous (e.g., lesions can progress and/or regress), studies on risk assessment tend to be complex, with a multitude of variables challenging the prediction at different times during the life of an individual [10]. For a clinician, the concepts of assessment of risk and prognosis are an important part of clinical decision making, individualized counseling, and anticipatory guidance.

In addition, risk factors may vary based on race, culture, and ethnicity [16–21]. Unfortunately, there are very few high-quality, longitudinal caries risk studies focusing on infants and toddlers [10, 22]. Furthermore, existing studies have been conducted primarily in selective populations in Northern Europe [23–29], diminishing the generalizability of these results to the US population. One recent study has been conducted in a low SES, African-American U.S. community [30]. In addition, Gao et al. [31] have recently suggested that practical biopsychosocial caries risk models without biological markers, such as the one tested here, are effective (sensitivity/specificity was 82%/73%) and promise to be cost-effective to reach children in a variety of settings.

Others have studied dental habits, attitudes, and beliefs in a range of settings. However, these studies have drawbacks in application to caregivers of toddlers due to the populations studied (age, race/ethnicity, and/or geographic location) and due to the range of topics covered. Dental habits, beliefs, and attitudes have been studied in adults [32–40], finding variability in beliefs and attitudes which may affect their own dental outcomes, but was not necessarily examined in the context of adults who were caring for toddlers. In parents of young children in Great Britain, knowledge and attitudes were found to vary due to education, ethnicity, and area of residence [41]. Dental knowledge, attitudes, and practices may also be impacted by the overall health of the child. Research has shown beliefs were found to differ between parents of children with and without congenital heart disease [42]. Relationships of caries in 3-year olds in Japan with child-rearing behaviors and mother's health behaviors were examined [43], finding a stronger association with the child-related behaviors than the mother's behaviors.

The purpose of this study was to evaluate how known caries risk factors evaluated longitudinally in young U.S. children differ by the ethnicity, income, and education of the caregiver. These factors had been identified through previous research as possible risk factors and were included as part of a one-year longitudinal risk study. If differences were found in the risk factors, as expected, this could indicate the need to target caregiver/patient education and preventive care intervention strategies based on the characteristics of the population or individual.

## 2. Methods

The study population included caregiver-toddler pairs in Indianapolis and Connersville, Indiana, USA. Subjects were recruited through four sites: (1) a primary-care-based study-recruitment system affiliated with a large metropolitan hospital serving a generally underserved and lower-income population, (2) the Oral Health Research Institute of the Indiana University School of Dentistry, (3) the Hispanic Center of Indianapolis, and (4) the rural town of Connersville. At sites 2–4 above, recruitment was done by radio and newspaper advertisements, as well as contacting an IRB-approved database of people who had participated in previous studies with us at those locations. The adult accompanying the child was required to self-identify as being the primary caregiver for the child. We defined “primary caregiver” (PCG) as the individual consistently responsible for the housing, health, and safety of the child. Toddlers ranged in age from 16 to 36 months at the time of recruitment, and were generally healthy based on the caregivers' responses to a medical history questionnaire. The study protocol, letter of informational consent, and other supporting documents were approved by the Indiana University Medical Center Institutional Review Board prior to their use. Written informed consent was obtained from all PCGs (and parent/legal guardian if different from the child's PCG) prior to their enrollment.

A caries risk questionnaire was developed to include questions related both to the PCG and the child regarding social, cultural, functional, psychological, sociodemographic, dietary, and biological factors that may affect transmission, development of caries, and access to care in these populations. Many of the questions were taken or modified from other risk assessment questionnaires and tools. An external review panel, which ranged from practitioners (pediatric dentists and pediatric physicians) to experts in the area of cariology, predictive modeling, and behavioral science, were provided a copy of the questionnaire and asked to review/edit the questionnaire to ensure that the initial draft of the questionnaire was reasonable in scope and that no established risk indicator had been omitted. After receiving separate IRB approval, the draft questionnaire was tested in a panel of 25 caregivers (nearly equal numbers of English and Spanish speaking), similar to the target population (had to consent to participate and have a child between 18 and 36 months of age), to ensure that the questions that were asked were worded appropriately for nonprofessionals, to eliminate jargon, to define or eliminate confusing terminology (e.g., words such as frequent, often, etc.), to ensure use of culturally-sensitive language, to finalize the organization of the items, and to verify the consistency of the structure of similar items. In most cases, it was believed that the majority of persons to be interviewed as PCG would be the mothers, but others (e.g., grandmothers, fathers) were to be included if it was found that they were responsible for providing the largest percentage of the child's care. Based on the results of the focus group data, some changes were made in the wording of questions, some questions were eliminated and some were reordered, and the questionnaire was finalized.

The final version of the questionnaire, which included 105 items (see Appendix), was administered by study personnel to the PCG (*n* = 396) using a multiple choice format, with responses recorded directly into a web-based database system. The caregiver chose whether to use the English or Spanish version of the questionnaire. Topics included in the questionnaire were categorized into: demographics, access to care, possible routes for oral bacteria transmission, usual dental and medical health practices of the caregiver and the toddler, dental beliefs of the caregiver, and snacking and drinking habits of the caregiver and the toddler. In addition, a subset of the caregivers (*n* = 250) was invited to participate in an additional investigation of health literacy. After additional informed consent, caregivers were administered the Short Test of Functional Health Literacy in Adults (S-TOFHLA) [44], with the caregiver given the option of using either the English or Spanish version.

The associations of PCG education and household income with race/ethnicity were tested using ANOVA, and Spearman correlation coefficients were calculated to measure the association between PCG education and household income. For the analyses, education levels were collapsed into 8th grade or lower, some high school, completed high school, some college, 4-year college, and postgraduate. We analyzed each survey item individually to assess the need to modify caregiver/patient education and preventive care intervention strategies based on demographic factors. To examine the associations of individual survey items (dependent variables in separate models) with the caregiver's race/ethnicity, the caregiver's education, and the household income simultaneously (three independent variables), multivariable logistic and linear regression analyses were used for survey items with qualitative responses and quantitative responses, respectively; thus race/ethnicity comparisons are adjusted for income and education, income comparisons are adjusted for race/ethnicity and education, and education comparisons are adjusted for race/ethnicity and income. *P*-values presented for the race/ethnicity comparisons are for the overall tests for any effect among the three groups; individual pairwise results are presented when significant but the *P*-values are not provided. Odds ratios presented for education and income are for a one-level change in the response categories. A 5% significance level was used for all analyses; although a large number of tests were performed, we did not adjust for multiple testing. A less restrictive cutoff without a multiple-testing adjustment provides a larger pool of possible differences that can be targeted when revising caregiver/patient education and preventive care intervention strategies.

## 3. Results

The study enrolled 396 caregiver-toddler pairs at baseline (two additional pairs were screened but did not qualify due to the child's medical condition), which is estimated to be approximately 70% of those invited to participate. Nearly all of the primary caregivers (378) were the child's mother, with the remaining caregivers consisting of 14 fathers, 2 grandmothers, 1 aunt, and 1 other. The caregivers' ages ranged from 18 to 64 years, with an average age of 28 (SD = 6) years. The children ranged in age from 16 to 36 months, with a mean of 26 (SD = 6) months, and ages did not differ significantly by race/ethnicity, income, or education of the caregiver. 51% of the toddlers were female. One hundred seventy-five (44%) of the caregivers self-identified themselves as Non-Hispanic African-American, 141 (36%) were Non-Hispanic White, 75 (19%) were Hispanic (all races), and 5 (1%) did not fall into one of the previous three categories.

Nearly one-third of Hispanic caregivers reported difficulty understanding information they receive from physicians and dentists, while the rate was less than ten percent in Non-Hispanic African-Americans and Non-Hispanic Whites (Table 1). Concurrently, caregivers with less education also were more likely to report these difficulties. Furthermore, health literacy, collected on a subset of 250 caregivers, was not different among race/ethnicity groups but was weakly associated with education (*r* = 0.18, *P* = .02). Non-Hispanic Whites were more likely to use city water as their primary drinking water source as opposed to bottled or well water. Interestingly, drinking water source was not related to income or education in this cohort. There was a moderately high correlation between education and income (*r* = 0.56, *P* = .0001) and moderate correlations for caregiver age with education (*r* = 0.42, *P* = .0001) and income (*r* = 0.38, *P* = .0001).

**Figure 1:**
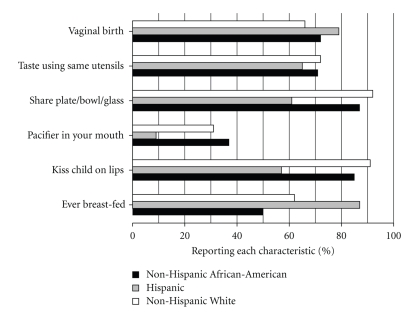
Relationships of race/ethnicity with oral bacteria transmission questionnaire responses (% responding “Yes”). Analyses were performed for each questionnaire item using multivariable logistic regression models with race/ethnicity, income, and education as predictors. Differences among race/ethnicity groups were found for “ever breast fed” (*P* = .0004, highest for Hispanics), “kiss child on lips” (*P* = .0001, lowest for Hispanics), “share plate/bowl/glass” (*P* = .0001, lowest for Hispanics), and “pacifier in your mouth” (*P* = .0156, lowest for Hispanics).

Habits of the caregivers that might lead to transmission of bacteria to the toddler differed by race/ethnicity (Figure 1), education, and income. Hispanic caregivers were less likely than Non-Hispanic African-American and Non-Hispanic White caregivers to put the toddler's pacifier in their own mouth (12% versus 37% and 31%, *P* = .0156), which was also associated with higher education (odds ratio 1.3, 95% CI 1.0–1.7, *P* = .0212) but not with income (odds ratio 1.0, 95% CI 0.8–1.1, *P* = .44). Tasting the child's food or drink using the same fork/spoon or glass was common in all race/ethnicity groups (approximately 70%, *P* = .87), but was more common with those reporting a higher income (odds ratio 1.3, 95% CI 1.1–1.4, *P* = .0013). Sharing food with the child using the same bowl/plate/glass and kissing the child on the lips occurred with nearly all Non-Hispanic African-American and Non-Hispanic White caregivers but was less frequent among Hispanics (*P* = .0001) and was more common with higher income (odds ratio 1.3, 95% CI 1.1–1.6, *P* = .0028). However, 87% of Hispanics ever breast-fed compared to 50% of Non-Hispanic African-Americans and 62% of Non-Hispanic Whites (*P* = .0004); breast-feeding was also more common with higher education (odds ratio 1.6, 95% CI 1.2–2.1, *P* = .0004) and higher income (odds ratio 1.1, 95% CI 1.0–1.3, *P* = .0458). 

Although caregivers with more education more often reported that their child had a dentist (Table 2), there were no differences in whether the child had ever been to the dentist. Because the toddlers may have similar access to care as their caregivers, the questionnaire also asked about dentist and physician visits made by the caregiver. Seventy-one percent of Non-Hispanic White caregivers, 53% of Non-Hispanic African-American caregivers, and 29% of Hispanic caregivers had a dentist (Table 1), and having a dentist was also associated with higher education attainment and higher income. Approximately half of Non-Hispanic African-Americans caregivers reported going to the dentist for regular checkups, while nearly 40% of Hispanic caregivers reported never going to the dentist. Interestingly, higher income was associated with caregivers going to the dentist for checkups, while lower education but not income was associated with never going to the dentist. In addition, patterns of caregiver visits to the physician differed by race/ethnicity (Table 1) but were not as affected by income or education, where only regular visits to the physician were associated with higher income.

**Figure 2:**
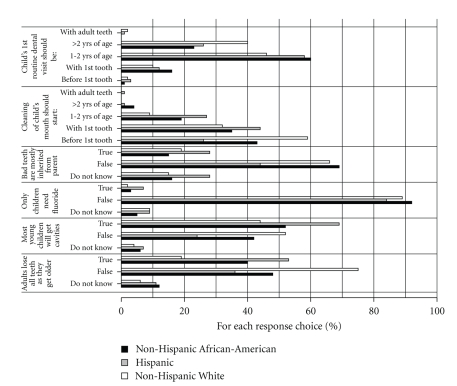
Relationships of race/ethnicity with dental beliefs of caregivers. Analyses were performed for each questionnaire item using multivariable logistic regression models with race/ethnicity, income, and education as predictors. Differences among race/ethnicity groups were found for “cleaning should start” (*P* = .0001, earlier for Non-Hispanic Whites), “bad teeth are mostly inherited from parent” (*P* = .0119, lower response of “false” for Hispanics), “most children will get cavities” (*P* = .0304, lower response of “false” for Hispanics), and “adults lose all teeth as they get older” (*P* = .0001, higher response of “false” for Non-Hispanic Whites).

Hispanic caregivers reported their children's teeth were brushed less frequently than teeth of Non-Hispanic African-Americans and Non-Hispanic Whites (Table 2). Caregivers with lower income were more likely to have problems with dry mouth when eating. Hispanic caregivers were more likely to be bothered by the appearance of their own teeth, which was not associated with education or income. Flossing was associated with more education but not with income or race/ethnicity. While there were differences among the race/ethnicity groups in how the caregivers felt about their child's and their own dental and medical health, education and income were generally not related to these ratings. Beliefs and knowledge (Figure 2) differed by race/ethnicity—adults eventually losing all their teeth (*P* = .0001, higher response of “false” for Non-Hispanic Whites), most children getting cavities (*P* = .0304, lower response of “false” for Hispanics), bad teeth being mostly inherited from parents (*P* = .0119, lower response of “false” for Hispanics), and when tooth cleaning should start (*P* = .0001, earlier for Non-Hispanic-Whites), with also a trend for when the child's first dental visit should be (*P* = .06, earliest for Non-Hispanic African-Americans and latest for Non-Hispanic Whites). Belief that adults will eventually lose all their teeth was associated with less education (odds ratio 1.4, 95% CI 1.1–1.8, *P* = .0072) and lower income (odds ratio 1.1, 95% CI 1.0–1.3, *P* = .0376), and belief that most children will eventually get cavities was associated with less education (odds ratio 1.3, 95% CI 1.0–1.6, *P* = .0258), while none of the other beliefs/knowledge assessed were significantly associated with education or income. 

Hispanic toddlers were more likely drink from a bottle (29%) compared to Non-Hispanic Whites toddlers (11%) and Non-Hispanic African-American toddlers (4%), while Non-Hispanic White toddlers and Non-Hispanic African-American toddlers were not significantly different. Non-Hispanic African-American toddlers were also less likely to drink from a sippy cup (67%) compared to Non-Hispanic Whites (84%) and Hispanic (87%) toddlers, who were not significantly different from each other (Table 3). Hispanic children were most likely to receive a bottle or sippy cup at bedtime or naptime. Although Hispanic caregivers cleaned their child's teeth after removing the drink more frequently than Non-Hispanic African-Americans or Non-Hispanic Whites, cleaning the child's teeth after removing the drink was rare for all races. Less than half of Hispanic children regularly sipped on drinks between meals, while nearly all Non-Hispanic African-American and Non-Hispanic White children did. Types of snacks and drinks usually eaten/drank between meals varied considerably among race/ethnicity groups for toddlers (Table 3) and for PCGs (Table 4), while snacking and between-meals drinks were typically not associated with education or income, with a specific exception of nondiet soda being associated with less education.

## 4. Discussion

Despite a decrease in dental caries prevalence in permanent teeth for most Americans since the early 1970s, oral health disparities remain across some population groups, and dental caries is still the most prevalent chronic disease of childhood [1]. Furthermore, between 1988–1994 and 1999–2004, caries experience in primary teeth of children aged 2–5 years has significantly increased from 24% to 28%, primarily due to an increase in the percent with fillings [45]. Unfortunately, as mentioned earlier, our current understanding of caries risk and etiological factors derived from longitudinal studies in young children in the United States is limited. Available caries risk questionnaire tools are, for the most part, expert-based tools. Examples include the Caries Risk Tool of the American Academy of Pediatric Dentistry [46], the ADA's Caries Risk Tool for children younger than 6 [47], and the Caries Management by Risk Assessment (CAMBRA) tool for children younger than 6 [48, 49]. While other studies have identified caries risk factors in low-SES rural [50] and low-SES African-American [30] communities, the prevalence of the risk factors may affect both the disease prevalence and the types of interventions that may be effective in preventing and/or treating caries. Age, socioeconomic status, and race/ethnicity differences as well as in non-US populations studied previously provided individual risk factor prevalence estimates, but only indirect evaluations of the effects of the sociodemographic factors on the risk factors could be made. In the present study, multiple factors from the caries risk questionnaire within the access to care, oral bacterial transmission, dental and medical health practices of the caregiver and the toddler, and snacking and drinking habits of the caregiver and the toddler areas were directly compared and differed by race/ethnicity, income, and/or education. Having general and pediatric dentists understand that these differences exist is only a first step. The information must be incorporated in improved strategies to treat and/or prevent caries in toddlers. 

With the limited sample size and single location sampled in this study, it is difficult to differentiate the effects of cultural influences, health knowledge gained through educational background, and income-based health utilization disparities on the risk factors; in other words, we were unable to look at the influence of interactions among the three factors or stratify the analyses. And while the study included three race/ethnicity groups, the single location of the study (Indiana) may not fully represent responses nationwide. A larger multisite study would be needed for increased generalizability as well as provide the sample size needed to differentiate among the cultural, income, and education influences on the risk factors. A large number of risk factors were examined, based on the extensive list of factors proposed or identified previously. Some of the risk factors differing by sociodemographic factors are likely to be false positives. Nevertheless the information from our study can provide useful risk factor prevalence data when revising caregiver/patient education and preventive care intervention strategies.

As mentioned above, our sample size was not large enough to justify a detailed examination of the 3-way interaction among race/ethnicity, income, and education to differentiate the effects of cultural influences, health knowledge gained through educational background, and income-based health utilization disparities on the risk factors. Regardless of the underlying “cause”, as others have suggested based on observations in various populations [32, 36], education and intervention strategies can be targeted generally to the population seen in the practice and specifically to individual patients. It is noteworthy to mention efforts in this country by medical (e.g., American Academy of Pediatrics and American Medical Association [51, 52]) and dental (e.g., American Dental Association [47], American Academy of Pediatric Dentistry [46, 53]) associations, among others, to stress not only the importance of a dental home early in life, but also the importance of risk-based preventive interventions and anticipatory guidance provided in a variety of settings to reach young children. In fact, a variety of programs have evolved in different places around the country. The “Into the Mouth of Babes” (IBM) program in North Carolina is one of the best examples of the effort resulting from the partnership between dentists and pediatricians to improve the oral health of children. The IMB program was initiated in 2000 and has led to a substantial increase in access to preventive dental services by enabling Medicaid children younger than 3 years of age to receive dental screening, counseling, and fluoride varnish in physicians' offices [54]. More work will certainly be needed to evaluate the acceptability and effectiveness of education and intervention strategies in targeted populations. 

One problem hindering treatment and prevention of caries in high-risk children is that they may not seek care from dentists regularly, if at all. Despite the importance of establishing a “dental home” in the first year of life, most children do not receive a dental examination, nor do the parents receive needed education on oral health [55]. This is especially true for those at the highest risk. While 89% of infants and one-year-olds have been examined by a physician, only 1.5% has had a dental appointment [53]. Some of the factors identified above could be included in discussions of “healthy behaviors” with the caregivers at well-child checkups. Patient education materials could also be developed to be made available through pediatrician and family practice offices. The results from our study may be useful to future investigators to focus the materials on factors prevalent in specific offices, such transmission of bacteria through sharing drinks or foods in higher income practices and providing drinks at bedtime or naptime in offices that have a high proportion of Hispanics. 

In conclusion, significant differences were found in all areas of the questionnaire related to race/ethnicity, income, and/or education. A larger followup study may be able to explore more detailed differentiation of the effects of cultural influences, health knowledge gained through educational background, and income-based health utilization disparities on the risk factors. Patient education and preventive care intervention studies may need to be targeted based on the characteristics of the population to achieve increase effectiveness.

## Figures and Tables

**Table 1:** Relationships of race/ethnicity, education, and income with questionnaire responses for demographics and access to care. Association of PCG education with race/ethnicity was tested using ANOVA. Remaining analyses were performed for each questionnaire item using multivariable logistic regression models with race/ethnicity, income, and education as predictors. Race/ethnicity *P*-values are for the overall test of any difference among the three groups. Questionnaire item numbers are listed in the left-most column (see the appendix for questionnaire). *N*(%) for race/ethnicity, odds ratio (OR) for Education and Income. NH-AA = Non-Hispanic African-American, NH-W = Non-Hispanic White.

		PCG race/ethnicity				PCG education		PCG income	
		Hispanic (*n* = 75)	NH-AA (*n* = 175)	NH-W (*n* = 141)	*P*-value	OR	*P*-value	OR	*P*-value
Q83	PCG education^a^				.0001				
	Less than high school	13 (17%)	0 (0%)	3 (2%)					
	1–3 years high school	20 (27%)	33 (19%)	20 (14%)					
	4 years high school	22 (29%)	66 (38%)	45 (32%)					
	1–3 years college	12 (16%)	46 (26%)	35 (25%)					
	4 years college	6 (8%)	21 (12%)	28 (20%)					
	Postgraduate	2 (3%)	9 (5%)	10 (7%)					
Q94	Difficult to understand info from dentist/MD	23(31%)	7(4%)	10(7%)	.0025	0.4	.0001	1.0	.8900
Q34	City drinking water	33(44%)	92(53%)	99(70%)	.0006	1.0	.8273	0.9	.2416
Q17	Child has a dentist	21(28%)	61(35%)	56(40%)	.2266	1.3	.0200	1.0	.4234
Q18	Child has been to a dentist	16(21%)	49(28%)	28(20%)	.2657	1.1	.6861	0.9	.5401
Q46	Child to physician if only if in pain/sick	7(9%)	29(17%)	28(20%)	.1369	0.9	.5682	1.1	.4226
Q74	PCG has a dentist	22(29%)	92(53%)	100(71%)	.0003	1.4	.0062	1.2	.0008
Q47	PCG to dentist for regular checkups	20(27%)	74(42%)	76(54%)	.0998	1.2	.0674	1.2	.0030
Q47	PCG never to dentist	29(39%)	30(17%)	15(11%)	.0047	0.6	.0065	0.9	.2742
Q48	PCG to physician if only if in pain/sick	31(41%)	55(31%)	78(55%)	.0001	1.2	.2364	0.9	.1303
Q48	PCG to physician for regular checkups	41(55%)	132(75%)	73(52%)	.0001	0.8	.0776	1.2	.0186

^a^The comparison of PCG education levels among race/ethnicity groups was not adjusted for income.

**Table 2:** Relationships of race/ethnicity, education, and income with questionnaire responses for dental and medical health practices of the primary caregiver (PCG) and the toddler. Analyses were performed for each questionnaire item using multivariable logistic or linear regression models with race/ethnicity, income, and education as predictors. Race/ethnicity *P*-values are for the overall test of any difference among the three groups. Questionnaire item numbers are listed in the left-most column (see the appendix for questionnaire). *N*(%) or Mean(SD) for race/ethnicity, odds ratio (OR) or correlation (R) for education and income. NH-AA = Non-Hispanic African-American, NH-W = Non-Hispanic White.

		PCG Race/Ethnicity				PCG Education		PCG Income	
		Hispanic (*n* = 75)	NH-AA (*n* = 175)	NH-W (*n* = 141)	*P*-value	OR/R	*P*-value	OR/R	*P*-value
Q6	PCG helps child brush	71(95%)	167(95%)	128(91%)	.0649	1.2	.5586	1.1	.2882
Q8	Child uses fluoride toothpaste	34(45%)	82(47%)	62(44%)	.0846	0.9	.5281	1.0	.9273
Q9	PCG checks child for cavities	53(71%)	85(49%)	71(50%)	.0337	0.7	.0046	0.9	.4050
Q36	Start Brush for 1st Tooth	13(17%)	46(26%)	53(38%)	.0243	1.1	.3197	1.0	.6055
Q37	Frequency of child's brushing^a^	2.3(1.2)	1.9(0.8)	1.8(0.9)	.0055	−0.09	.9872	−0.07	.2822
Q38	Frequency of PCG's brushing^a^	1.3(0.5)	1.5(0.7)	1.4(0.6)	.0611	−0.04	.9771	−0.10	.2039
Q39	Frequency of PCG's flossing^a^	4.3(1.7)	4.1(1.7)	3.7(1.5)	.4248	−0.25	.0030	−0.23	.0731
Q49	Child's dental health^b^	2.8(1.1)	2.1(1.0)	2.0(0.9)	.0001	−0.15	.3530	−0.12	.4345
Q50	Taking care of child's dental health^b^	3.1(0.9)	2.4(1.0)	2.5(0.9)	.0001	−0.01	.2670	0.00	.6502
Q51	Child's medical health^b^	2.1(0.9)	1.6(0.8)	1.7(0.7)	.0003	−0.08	.9636	−0.07	.2382
Q52	Taking care of child's medical health^b^	2.2(0.9)	1.4(0.6)	1.6(0.7)	.0001	−0.12	.3430	−0.05	.7470
Q53	PCG's dental health^b^	3.9(0.8)	3.5(1.1)	3.3(1.1)	.0906	−0.32	.0059	−0.35	.0001
Q54	Taking care of own dental health^b^	3.6(0.8)	3.2(1.1)	3.0(1.0)	.0285	−0.15	.1470	−0.15	.2572
Q55	PCG's medical health^b^	3.1(0.9)	2.3(1.0)	2.5(0.9)	.0001	−0.17	.0643	−0.09	.9939
Q56	Taking care of own medical health^b^	2.9(1.0)	2.4(1.1)	2.6(0.9)	.0033	−0.05	.4921	−0.11	.0357
Q69	PCG often has dry mouth when eating	10(13%)	34(19%)	23(16%)	.4116	0.9	.3456	0.8	.0137
Q70	PCG has restorations for past cavities	57(76%)	118(67%)	123(87%)	.0010	1.7	.0008	1.0	.6392
Q71	PCG has current cavities	42(56%)	79(45%)	47(33%)	.1029	0.8	.0494	0.9	.2353
Q72	PCG bothered by how own teeth look	46(61%)	79(45%)	57(40%)	.0309	0.9	.3523	1.0	.5640
Q73	PCG needs dental treatment now	64(85%)	123(70%)	78(55%)	.0009	0.9	.3433	0.9	.1089
Q75	PCG scared of going to the dentist	15(20%)	50(29%)	29(21%)	.0404	0.8	.1557	1.0	.9971
Q77	PCG uses fluoride toothpaste	56(75%)	154(88%)	118(84%)	.2974	1.2	.2525	1.0	.9745

^a^Frequency rated on a 1–6 scale: 1 = more than once per day, 2 = once per day, 3 = several times a week, 4 = several times a month, 5 = a few times a year, and 6 = never.^b^Ratings on a 1–5 scale: 1 = excellent, 2 = very good, 3 = good, 4 = fair, and 5 = poor.

**Table 3:** Relationships of race/ethnicity, education, and income with questionnaire responses for snacking and drinking habits of the toddler. Analyses were performed for each questionnaire item using multivariable logistic or linear regression models with race/ethnicity, income, and education as predictors. Race/ethnicity *P*-values are for the overall test of any difference among the three groups. Questionnaire item numbers are listed in the left-most column (see the appendix for questionnaire). *N*(%) or Mean(SD) for race/ethnicity, odds ratio (OR) or correlation (R) for education and income. NH-AA = Non-Hispanic African-American, NH-W = Non-Hispanic White.

		PCG Race/Ethnicity				PCG Education		PCG Income	
		Hispanic (*n* = 75)	NH-AA (*n* = 175)	NH-W (*n* = 141)	*P*-value	OR/R	*P*-value	OR/R	*P*-value
Q1	Child usually drinks from a bottle	22(29%)	7(4%)	15(11%)	.0001	1.1	.2559	1.1	.1013
Q2	Child usually drinks from a sippy cup	65(87%)	118(67%)	118(84%)	.0001	0.6	.0307	0.7	.1374
Q40	Frequency of bottle/sippy at sleep time^a^	2.9(2.1)	4.5(2.0)	4.4(2.0)	.0001	0.15	.7376	0.16	.0137
Q42	Brushing frequency after sleep-time drinks^a^	4.6(2.0)	5.9(1.6)	5.7(1.9)	.0001	0.19	.6731	0.19	.0075
Q26	Child regularly sips drinks between meals	32(43%)	168(96%)	129(91%)	.0001	0.8	.1461	1.0	.6605
Q31	Child usually snacks on candy	31(41%)	72(41%)	36(26%)	.0557	0.8	.1294	0.9	.4255
Q31	Child usually snacks on cookies	46(61%)	104(59%)	63(45%)	.0266	0.8	.1237	1.0	.6380
Q31	Child usually snacks on fresh fruit	70(93%)	145(83%)	108(77%)	.0035	1.2	.1935	1.1	.2418
Q31	Child usually snacks on cake	23(31%)	35(20%)	7(5%)	.0012	0.7	.0480	0.9	.6211
Q31	Child usually snacks on ice cream	39(52%)	47(27%)	24(17%)	.0001	1.0	.9244	0.9	.3391
Q31	Child usually snacks on cereal with milk	44(59%)	93(53%)	50(35%)	.0019	0.9	.2473	1.0	.6969
Q31	Child usually snacks on dried fruit	22(29%)	53(30%)	45(32%)	.9827	1.0	.8352	1.0	.8686
Q31	Child usually snacks on popcorn	27(36%)	71(41%)	52(37%)	.8911	1.1	.8031	1.0	.8911
Q31	Child usually snacks on chips	45(60%)	127(73%)	65(46%)	.0001	0.9	.5032	0.9	.0442
Q31	Child usually snacks on dry cereal	24(32%)	103(59%)	89(63%)	.0002	1.1	.2060	1.0	.8281
Q31	Child usually snacks on yogurt	47(63%)	59(34%)	64(45%)	.0002	1.0	.9864	1.2	.0120
Q33	Child usually drinks water between meals	65(87%)	119(68%)	98(70%)	.0028	1.1	.3031	1.0	.6363
Q33	Child usually drinks nondiet soda between meals	22(29%)	16(9%)	14(10%)	.0038	0.7	.0403	0.9	.4546
Q33	Child usually drinks juice between meals	67(89%)	146(83%)	98(70%)	.0012	1.0	.6801	0.9	.4027
Q33	Child usually drinks sugared fruit drink between meals	46(61%)	48(27%)	31(22%)	.0001	0.8	.0296	1.0	.6489
Q33	Child usually drinks milk between meals	63(84%)	130(74%)	109(77%)	.2159	1.2	.2507	0.9	.2117
Q44	Frequency child drinks tap water^a^	3.2(2.3)	2.6(1.9)	1.8(1.5)	.0001	–0.03	.6840	–0.02	.8132

^a^Frequency rated on a 1–6 scale: 1 = more than once per day, 2 = once per day, 3 = several times a week, 4 = several times a month, 5 = a few times a year, and 6 = never.

**Table 4:** Relationships of race/ethnicity, education, and income with questionnaire responses for snacking and drinking habits of the primary caregiver (PCG). Analyses were performed for each questionnaire item (items 80 through 82—see the appendix for questionnaire) using multivariable logistic regression models with race/ethnicity, income, and education as predictors. Race/ethnicity *P*-values are for the overall test of any difference among the three groups. *N*(%) for Race/Ethnicity, Odds Ratio (OR) for education and income. NH-AA = Non-Hispanic African-American, NH-W = Non-Hispanic White.

	PCG race/ethnicity				PCG education		PCG income	
	Hispanic (*n* = 75)	NH-AA (*n* = 175)	NH-W (*n* = 141)	*P*-value	OR	*P*-value	OR	*P*-value
PCG has snacks on most days	48(64%)	143(82%)	108(77%)	.0314	1.4	.0144	1.1	.3236
PCG usually snacks on candy	16(21%)	77(44%)	37(26%)	.0001	0.9	.4449	1.0	.7326
PCG usually snacks on cookies	27(36%)	81(46%)	49(35%)	.0140	1.0	.7664	1.1	.4027
PCG usually snacks on fresh fruit	46(61%)	98(56%)	69(49%)	.1863	1.2	.0874	1.0	.4175
PCG usually snacks on cake	21(28%)	52(30%)	13(9%)	.0002	0.9	.5992	1.0	.8133
PCG usually snacks on ice cream	25(33%)	59(34%)	28(20%)	.0143	1.1	.7390	1.0	.5263
PCG usually snacks on popcorn	15(20%)	73(42%)	47(33%)	.0111	1.1	.3649	1.1	.0372
PCG usually snacks on chips	24(32%)	118(67%)	57(40%)	.0001	1.1	.5857	1.0	.7557
PCG usually drinks water between meals	69(92%)	135(77%)	98(70%)	.0003	1.2	.3087	1.1	.0777
PCG usually drinks nondiet soda between meals	28(37%)	94(54%)	62(44%)	.0186	0.7	.0105	1.0	.4225
PCG usually drinks diet soda between meals	11(15%)	18(10%)	27(19%)	.2055	1.4	.0381	1.1	.2771
PCG usually drinks juice between meals	44(59%)	89(51%)	31(22%)	.0001	0.8	.0771	1.0	.4755
PCG usually drinks sugared fruit drink	36(48%)	45(26%)	17(12%)	.0001	0.9	.8146	0.9	.3596
PCG usually drinks milk between meals	35(47%)	41(23%)	40(28%)	.0054	0.9	.4149	0.9	.1142
PCG usually drinks tea between meals	8(11%)	32(18%)	45(32%)	.0006	1.0	.7849	0.9	.2744
PCG usually drinks coffee w/sugar between meals	21(28%)	12(7%)	13(9%)	.0001	0.9	.5356	1.1	.3277

## Appendix

### Questionnaire

*First, I'd like to ask about the child's (i.e., refers to the child in this study) eating and health habits*. Please answer yes or no for each of the following questions.

1. Does the child usually drink from a bottle?
  Yes
  No
2. Does the child usually drink from a sippy cup?
  Yes
  No
3. Is the child currently being breast-fed?
  Yes
  No
4. If not currently breast-fed, was the child ever breast-fed? (if yes, how long was the child breastfeed for: _ _ _ _ _ _ months)
  Yes
  No
5. Does the child share a toothbrush with anyone? (if yes, indicate who: _ _ _ _ _ _)
  Yes
  No
  Does not use one
6. Do you help the child brush his or her teeth?
  Yes
  No
7. When you or the child brushes his/her teeth, do you use toothpaste?
  Yes
  No
8. Does the child's toothpaste have fluoride in it?
  Yes
  No
  Do not know
9. Do you ever check the child's teeth for cavities?
  Yes
  No
10. Does the child have cavities now?
  Yes
  No
  Do not know
11. Has the child had cavities or fillings in the past?
  Yes
  No
12. Has the child had teeth pulled because of cavities?
  Yes
  No
13. Have other children (brothers, sisters, or others) in the child's household had cavities or fillings?
  Yes
  No
  Only child
14. Does the child have problems chewing?
  Yes
  No
15. Does the child have a tooth that hurts?
  Yes
  No
16. Do you think the child is bothered about how his/her teeth look?
  Yes
  No
17. Does the child have a dentist?
  Yes
  No
18. Has the child ever been to the dentist?
  Yes
  No
19. Does the child use other products for his/her teeth (mouth rinse, prescription toothpaste, tablets, drops, or other) with fluoride? (do not count water)
  Yes
  No
  Do not know
20. Do or did you ever put the child's pacifier in your mouth before giving it to him/her?
  Yes
  No
  Does not use it
21. Do you ever kiss the child on the lips?
  Yes
  No
22. When the child was born, were you told by his/her doctor that his/her birth-weight was low?
  Yes
  No
  Do not know
23. Did the child's mother get prenatal care?
  Yes
  No
  Do not know
24. Do you ever taste the child's food and/or drinks using the same spoon, fork, glass, or other?
  Yes
  No
25. Do you ever share food or drinks with the child from the same plate, bowl, or glass?
  Yes
  No
26. Does the child regularly sip on drinks between meals on most days?
  Yes
  No
27. Does the child use antibiotics more than every three months?
  Yes
  No
28. Does the child regularly use medications at bedtime or during the night?
  Yes
  No (skip to no. 30)
29. Do you regularly brush the child's teeth after use of the medication?
  Yes
  No
30. Does the child have snacks most days?
  Yes
  No (skip to no. 32)
31. What kinds of foods does he/she **usually** snack on? (A snack is food eaten in between regular meals.) Please read the list, and check all foods that apply.
  Candy
  cakes/cupcakes
  cereal with milk
  chips
  cookies
  crackers
  dried fruit (e.g., raisins)
  dry cereal
  fresh fruit
  ice cream
  popcorn
  yogurt
  other (list or specify: _ _ _ _ _ _)
32. How many regular meals (e.g., breakfast, lunch, dinner, or other) does the child eat per day? _ _ _ _ _ _
33. What does he/she **usually** drink with a snack or in between meals? Please read the list, and check all drinks that apply.
  water
  juice (100% juice)
  milk
  soda (with sugar)
  fruit drink (with sugar)
  tea
  soda (diet or sugar free)
  fruit drink (sugar-free)
  other (list or specify: _ _ _ _ _ _)
34. The child's main source of drinking water is:
  city
  bottled
  well
  other (list or specify: _ _ _ _ _ _)
35. The child got his/her first tooth at _ _ _ _ _ _ months of age.
36. When did brushing/cleaning of the child's teeth start? (check all that apply)
  when the first tooth came into the mouth
  younger than 12 months
  13–24 months
  25–36 months
  older than 36 months
  not brushing/cleaning teeth yet
37. How often do you or the child clean or brush the child's teeth? Would you say…
  More than once a day
  Once a day
  Several times a week
  Several times a month
  A few times a year
  Never
38. How often do you brush your own teeth?
  More than once a day
  Once a day
  Several times a week
  Several times a month
  A few times a year
  Never
39. How often do you floss your own teeth?
  More than once a day
  Once a day
  Several times a week
  Several times a month
  A few times a year
  Never
40. How often does the child get a bottle/sippy cup in bed, at either bedtime or naptime with something *other* than water in it?
  More than once a day
  Once a day
  Several times a week
  Several times a month
  A few times a year
  Never
41. How often does the child get a bottle/sippy cup filled with something *other* than water in it during the day (do not count mealtimes)?
  More than once a day
  Once a day
  Several times a week
  Several times a month
  A few times a year
  Never
42. How often do you clean your child's teeth after you remove the bottle/sippy cup at night (after going to bed)?
  More than once a day
  Once a day
  Several times a week
  Several times a month
  A few times a year
  Never
  Does not drink at night
43. How often does your child breast-feed at night?
  More than once a day
  Once a day
  Several times a week
  Several times a month
  A few times a year
  Never
  Does not breast-feed
44. How frequently does your child drink tap water or drinks prepared with tap water?
  More than once a day
  Once a day
  Several times a week
  Several times a month
  A few times a year
  Never
45. Which sentence or sentences below describe how you decide (or intend to decide) when to take **the child** to the dentist? (check all that apply)
  I only take the child to the dentist if he/she has pain or a problem with his/her teeth.
  I take the child to the dentist regularly because he/she has problems with the teeth or gums.
  I take the child to the dentist for regular checkups.
  I do not take the child to the dentist as often as the dentist wants me to.
  I never take the child to the dentist.
46. Which sentence or sentences below describe how you decide when to take **the child** to the doctor? (check all that apply)
  I only take the child to the doctor if he/she has pain or is sick.
  I take the child to the doctor regularly because he/she has a health problem.
  I take the child to the doctor for regular checkups.
  I don't take the child to the doctor as often as the doctor wants me to.
  I never take the child to the doctor.
47. Which sentence or sentences below describe how you decide when to see **your** dentist? (check all that apply).
  I only go to the dentist if I have pain or if I have a problem with my teeth or gums.
  I see my dentist regularly because I have problems with my teeth or gums.
  I see my dentist for regular checkups.
  I don't see my dentist as often as the dentist wants me to.
  I never go to the dentist.
48. Which sentence or sentences below describe how you decide when to see **your** doctor? (check all that apply)
  I only go to the doctor if I have pain or if I'm sick.
  I see my doctor regularly because of a health problem.
  I see my doctor for regular checkups.
  I don't see my doctor as often as the doctor wants me to.
  I never go to the doctor.
49. How would you describe **the child's** dental (teeth and gums) health? Would you say it is…
  Excellent
  Very good
  Good
  Fair
  Poor
50. How would you describe how you take care of **the child's** dental (teeth and gums) health?
  Excellent
  Very good
  Good
  Fair
  Poor
51. How would you describe **the child's** medical health?
  Excellent
  Very good
  Good
  Fair
  Poor
52. How would you describe how you take care of **the child's** medical health?
  Excellent
  Very good
  Good
  Fair
  Poor
53. How would you describe **your** dental (teeth and gums) health?
  Excellent
  Very good
  Good
  Fair
  Poor
54. How would you describe how you take care of **your** dental (teeth and gums) health?
  Excellent
  Very good
  Good
  Fair
  Poor
55. How would you describe **your ** medical health?
  Excellent
  Very good
  Good
  Fair
  Poor
56. How would you describe how you take care of **your** medical health?
  Excellent
  Very good
  Good
  Fair
  Poor
57. How satisfied are you with **the child's **dentist/dental care?
  Very Satisfied
  Somewhat Satisfied
  Somewhat Dissatisfied
  Very Dissatisfied
  Not Applicable
58. How satisfied are you with **the child's **doctor/medical care?
  Very Satisfied
  Somewhat Satisfied
  Somewhat Dissatisfied
  Very Dissatisfied
  Not Applicable
59. How satisfied are you with **your** dentist/dental care?
  Very Satisfied
  Somewhat Satisfied
  Somewhat Dissatisfied
  Very Dissatisfied
  Not Applicable
60. How satisfied are you with **your **doctor/medical care?
  Very Satisfied
  Somewhat Satisfied
  Somewhat Dissatisfied
  Very Dissatisfied
  Not Applicable

*The next questions focus on your dental beliefs*. Please answer True or False to the following statements.

(61) Most adults will lose all their teeth as they get older
  True
  False
  Do not know
(62) Most young children will get cavities
  True
  False
  Do not know
(63) Only children need fluoride
  True
  False
  Do not know
(64) The type of food and drink a child eats or drinks may cause cavities
  True
  False
  Do not know
(65) Baby teeth are important to take care of
  True
  False
  Do not know
(66) Bad teeth are mostly inherited from the parents
  True
  False
  Do not know
(67) Cleaning of the mouth of a child should begin: (check all that apply)
  before the first tooth comes in
  as soon as the first tooth comes in
  12–24 months-of-age
  after 24 months-of-age
  when the adult teeth come in
  do not know
(68) A child's first routine dental visit should be: (check all that apply)
  before the first tooth comes in
  as soon as the first tooth comes in
  12–24 months-of-age
  after 24 months-of-age
  when the adult teeth come in
  do not know

The next questions focus on your own eating and health habits.

(69) Does your mouth often feel dry when you eat a meal?
  Yes
  No
(70) Have you had any cavities in the past, which are now restored/fixed?
  Yes
  No
(71) Do you have cavities now that are not “fixed”?
  Yes
  No
  Do not know
(72) Are you bothered by how your teeth look?
  Yes
  No
(73) Do you think you need dental treatment (other than a cleaning) now?
  Yes
  No
(74) Do you have a dentist?
  Yes
  No
(75) Are you scared of going to the dentist?
  Yes
  No
(76) When you brush your teeth, do you use toothpaste?
  Yes
  No
(77) Does the toothpaste you use have fluoride in it?
  Yes
  No
  Do not know
(78) Do you use other products for your teeth (mouth rinse, prescription toothpaste, other) with fluoride?
  Yes
  No
  Do not know
(79) Have you had more than half of your adult teeth pulled?
  Yes
  No
(80) Do you have snacks most days?
  Yes
  No (skip to no. 82)
(81) What kinds of foods do you **usually** snack on? (A snack is food eaten in between regular meals.) Please read the list, and check all foods that apply.
  candy
  cakes/cupcakes
  cereal with milk
  chips
  cookies
  crackers
  dried fruit (e.g., raisins)
  dry cereal
  fresh fruit
  ice cream
  popcorn
  yogurt
  other (list or specify: _ _ _ _ _ _)
(82) What do you **usually** drink with a snack or in between meals? Please read the list, and check all drinks that apply.
  water
  juice (100% juice)
  milk
  coffee without sugar
  soda (with sugar)
  fruit drink (with sugar)
  tea
  coffee with sugar
  soda (diet or sugar-free)
  fruit drink (sugar-free)
  other (list or specify _ _ _ _ _ _)

Now we are going to ask some questions about you, the child's family, and the child.

(83) What is the highest grade in school you have completed?
  Grade school (1, 2, 3, 4, 5, 6, 7, 8)
  High school (9, 10, 11, 12)
  College (13, 14, 15, 16)
  Post graduate (17+)
(84) Does **the child** have Medicaid or Hoosier Healthwise?
  Yes
  No
(85) Does **the child** have health insurance? (Private Health insurance)
  Yes
  No
(86) Does **the child** receive free care (medical or dental) through any other program?
  Yes
  No
(87) Do **you** have health insurance?
  Yes
  No
(88) Do **you** have dental insurance?
  Yes
  No
(89) In what country were **you** born?
  U.S.
  Another country List: _ _ _ _ _ _
(90) In what country was **the child** born?
  U.S. (skip to 92)
  Another country List: _ _ _ _ _ _
(91) How long has the child been in the United States? Remember, all information you give us is confidential.
  _ _ _ _ _ _ years
(92) What language do you usually speak at home?
  English
  Another language List: _ _ _ _ _ _
(93) Because of language, do you have difficulty talking to the child's dentist or doctor?
  Yes
  No
  Regularly use an interpreter
(94) Is it sometimes difficult for you to understand the information given by the child's doctor or dentist?
  Yes
  No
(95) Do you consider **the child** to be Spanish, Hispanic, or Latino?
  Yes
  No
(96) Do you consider **yourself** to be Spanish, Hispanic, or Latino?
  Yes
  No
(97) What is **the child's** racial or ethnic background? (Mark one or more races to indicate the race or races you consider the child to be)
  White or Caucasian
  African American or Black (Black refers to people with ancestors from Sub-Saharan Africa, the West Indies, the Caribbean (including Haiti, Jamaica, Barbados, and Cape Verde)
  Asian (specify subgroup _ _ _ _ _ _)
  Native Hawaiian or other Pacific Islander
  American Indian or Alaskan Native
  Other (specify: _ _ _ _ _ _)
(98) What is **your** racial or ethnic background? (Mark one or more races to indicate the race or races you consider yourself to be)
  White or Caucasian
  African American or Black (Black refers to people with ancestors from Sub-Saharan Africa, the West Indies, the Caribbean (including Haiti, Jamaica, Barbados, and Cape Verde)
  Asian (specify subgroup _ _ _ _ _ _)
  Native Hawaiian or other Pacific Islander
  American Indian or Alaskan Native
  Other (specify: _ _ _ _ _ _)
(99) How many adults live in the child's household?
  Number of Adults/Children _ _ _ _ _ _
(100) How many adults in the child's household have paid jobs?
  Number of Adults/Children _ _ _ _ _ _
(101) How many adults other than yourself take care of the child regularly?
  Number of Adults/Children _ _ _ _ _ _
(102) How many children live in the child's household, including the study child?
  Number of Adults/Children _ _ _ _ _ _
(103) Do you have a job?
  Yes
  No
(104) Do you have transportation to go to the doctor or dentist?
  Yes
  No
(105) Which of the following categories best represents the combined income for all family members in your household added together for the past 12 months? (Remember, all information is completely confidential). Please read the list of income categories and check the one that applies to you.
  Less than $5000
  $5,000–$9,999
  $10,000–$19,999
  $20,000–$29,999
  $30,000–$39,999
  $40,000–$49,999
  $50,000–$79,999
  $80,000–$99,999
  $100,000 or more
  Do not know
